# Basis for treatment of tuberculosis among HIV-infected patients in Tanzania: the role of chest x-ray and sputum culture

**DOI:** 10.1186/1471-2334-8-32

**Published:** 2008-03-06

**Authors:** Muhammad Bakari, Robert D Arbeit, Lillian Mtei, Johnson Lyimo, Richard Waddell, Mecky Matee, Bernard F Cole, Susan Tvaroha, C Robert Horsburgh, Hanna Soini, Kisali Pallangyo, C Fordham von Reyn

**Affiliations:** 1 Muhimbili University College of Health Sciences, Dar es Salaam, Tanzania; 2 Paratek Pharmaceuticals, Boston, MA, USA; 3 Infectious Disease and International Health, Dartmouth Medical School, Lebanon, NH, USA; 4 Boston University School of Public Health, Boston, MA, USA; 5 National Public Health Institute of Finland, Turku, Finland

## Abstract

**Background:**

Active tuberculosis (TB) is common among HIV-infected persons living in tuberculosis endemic countries, and screening for tuberculosis (TB) is recommended routinely. We sought to determine the role of chest x-ray and sputum culture in the decision to treat for presumptive TB using active case finding in a large cohort of HIV-infected patients.

**Methods:**

Ambulatory HIV-positive subjects with CD4 counts ≥ 200/mm^3 ^entering a Phase III TB vaccine study in Tanzania were screened for TB with a physical examination, standard interview, CD4 count, chest x-ray (CXR), blood culture for TB, and three sputum samples for acid fast bacillus (AFB) smear and culture.

**Results:**

Among 1176 subjects 136 (12%) were treated for presumptive TB. These patients were more frequently male than those without treatment (34% vs. 25%, respectively; p = 0.049) and had lower median CD4 counts (319/μL vs. 425/μL, respectively; p < .0001). Among the 136 patients treated for TB, 38 (28%) had microbiologic confirmation, including 13 (10%) who had a normal CXR and no symptoms. There were 58 (43%) treated patients in whom the only positive finding was an abnormal CXR. Blood cultures were negative in all patients.

**Conclusion:**

Many ambulatory HIV-infected patients with CD4 counts ≥ 200/mm^3 ^are treated for presumptive TB. Our data suggest that optimal detection requires comprehensive evaluation, including CXR and sputum culture on both symptomatic and asymptomatic subjects.

## Background

Tuberculosis (TB) is the major cause of death from AIDS in most areas of the developing world [1,2]. The high mortality of HIV-associated TB reflects multiple factors, including lack of access to care, delayed or missed diagnosis of TB and acceleration of HIV infection [2-5]. Active TB is considered an indication for anti-retroviral therapy (ART) in HIV-infected persons with CD4 counts <350, but treatment of co-infection may be complicated by drug interactions and by immune reconstitution syndrome [6,7]. For these reasons screening for active TB is recommended for patients with HIV living in TB-endemic regions. However, only limited data are available on the relative sensitivity of different methods of screening for active TB among HIV-infected patients with CD4 counts ≥ 200. The World Health Organization (WHO) recommends screening for TB prior to ART based on symptoms followed by sputum acid fast bacillus (AFB) smear; chest x-ray (CXR) and sputum culture are not routine in their method [6,8].

This report describes our experience applying a comprehensive screening program (including both CXR and sputum culture) to both symptomatic and asymptomatic ambulatory HIV-infected subjects with CD4 counts ≥ 200 being evaluated for a TB vaccine trial in Tanzania. With this approach, 12% of those screened were treated for presumptive TB based on the local standards of care. Fewer than half of these cases would have been detected by symptoms and AFB smear alone; many were only detected by CXR and some cases only by sputum culture. These observations have major implications for HIV programs.

## Methods

### Study subjects and protocol

Subjects are screened for HIV and tuberculosis as part of the DarDar (Dartmouth Medical School-Dar es Salaam) Study, a Phase III trial of a prime-boost vaccine strategy for the prevention of HIV-associated TB (prime = childhood BCG, boost = multiple dose whole cell inactivated mycobacterial vaccine) [9] being conducted in Tanzania. Ambulatory HIV-infected subjects age ≥ 18 years in apparent good health are referred from HIV voluntary counseling and testing (VCT) centers in Dar es Salaam or by other study subjects. Informed consent was obtained from all subjects.

Eligibility requirements for the vaccine study include two positive ELISA antibody tests for HIV, CD4 count ≥ 200/mm^3 ^and BCG scar. All subjects meeting these criteria were evaluated for active TB by symptoms, chest x-ray and microbiology. The latter involved collecting three expectorated sputum samples for AFB smear and culture. Patients were instructed to cough deeply and were encouraged to produce one spot sample and bring two subsequent first morning samples; sputum induction was not used. A single 10 mL mycobacterial blood culture was processed using the MB/BacT automated system (bioMerieux, Inc, Durham, NC, USA). A tuberculin skin test (TST) was performed with 0.1 mL intradermal RT-23 (State Serum Institute, Copenhagen) and read at 48–72 hrs as mm induration in the transverse diameter.

For the purposes of this report symptoms of TB were defined as either cough or fever for ≥ 2 weeks or both. Chest x-rays were read by the principal radiologist at the university teaching hospital, Muhimbili University College of Health Sciences (MUCHS); the requisition indicated whether the patient has suspect TB. An abnormal x-ray is defined as the presence of a focal infiltrate, cavity formation, hilar adenopathy, or a miliary pattern. The decision to treat for suspect TB was made by study physicians in collaboration with physicians from the Tanzania National Tuberculosis and Leprosy Program (NTLP) based on clinical, laboratory and/or radiologic features consistent with TB. CXR and AFB smear results were available for these decisions and patients were typically assessed for radiologic response to a 10 day course of antibiotic therapy for community acquired pneumonia before the decision to treat for TB. Other than blood culture, there was no detailed testing for extrapulmonary TB among the subjects described in the present report. Follow-up data on whether the subject started treatment as recommended was obtained from the NTLP.

In the DarDar Study, the endpoints of definite or probable TB are based on strict definitions (Table 1) which are applied by a panel of three experts who review the clinical, radiologic and microbiologic data. Among subjects without microbiologic confirmation, classification as definite or probable TB requires 6–8 months of follow-up to determine the response to TB treatment. The purpose of this report is to provide data on the basis for an initial clinical decision to treat for TB in accordance with local standards of care. The subjects treated for TB in the present report were discharged from the study without further follow-up and, consequently, we cannot confirm that all the treated patients (e.g, patients with symptoms alone) would meet strict research-oriented criteria for TB.

**Table 1:** Definitions of TB for the DarDar Study

Definite TB	1. One or more sputum cultures positive for *Mycobacterium tuberculosis *(MTB) with ≥ 10 colony forming units (CFU); or,
	2. Two or more sputum cultures with 1–9 CFU of MTB (indeterminate MTB culture); or,
	3. Two or more positive sputum smears for acid fast bacilli (AFB) *; or,
	4. One or more cultures for MTB from the blood or other sterile body site.
Probable TB	1. Positive chest x-ray plus either
	a. one positive sputum AFB smear, or,
	b. one indeterminate MTB culture result; or,
	2. Clinical symptoms/signs plus either
	a. one positive sputum AFB smear, or,
	b. an indeterminate MTB culture result; or,
	3. Clinical symptoms/signs and a positive x-ray plus a response to anti-TB therapy; or,
	4. One positive sputum AFB smear from a sterile site plus clinical symptoms/signs of tuberculosis; or,
	5. Caseous necrosis on a tissue biopsy.

AFB, acid fast bacillus; CFU, colony forming units; MTB, *Mycobacterium tuberculosis*; TB, tuberculosis.

* a positive AFB smear is defined as ≥ 2 acid-fast organisms per 100 oil immersion field

The research protocol was approved by the Ethics Committee of the Muhimbili University College of Health Sciences and the Dartmouth Committee for the Protection of Human Subjects.

### Laboratory methods

Serum samples are tested using 2 different HIV ELISA methods: Vironostika HIV Uni-Form II Ag/Ab (BioMerieux, Boxtel, The Netherlands) and Vironostika HIV Uni-Form II Plus O (BioMerieux, Boxtel, The Netherlands). Subjects reactive on both assays are considered HIV-infected. Blood is collected in EDTA tubes for enumeration of CD4 cells by FASCount (BD Biosciences, Franklin Lakes, NJ) after staining with monoclonal antibodies [10].

Sputum samples are examined by direct microscopy using auramine-rhodamine staining with confirmation of positives by Ziehl Neelsen AFB staining. Sputum samples are also decontaminated in 2% NaOH for 30 minutes, concentrated by centrifugation at 3000 g, examined again by microscopy and cultured on Lowenstein Jensen slants for 10 weeks at 37°C. Sputum culture contamination rates were approximately 1% during the period of this report.

Blood is cultured using the MB/BacT method according to manufacturer's instructions (Bio-Merieux, Lyon, France). Characteristic slow-growing mycobacteria are presumptively identified as *M. tuberculosis*. Putative *M. tuberculosis *isolates are shipped to Dartmouth to confirm *M. tuberculosis *complex by DNA probe testing (AccuProbe, Gen-Probe, San Diego, CA)

### Statistical analysis

Standard descriptive statistical methods are used to determine median, interquartile range and the percent of subjects with particular characteristics. All p-values are based on Fisher's exact test or Wilcoxon test, as appropriate, and are 2-sided. A p-value < 0.05 is considered statistically significant.

## Results

**Figure 1:**
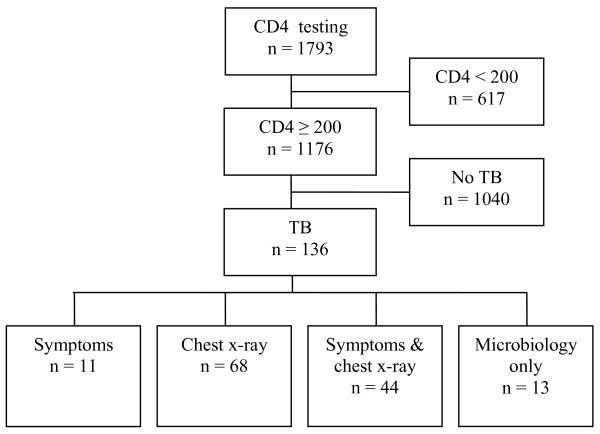
**Basis for TB treatment in HIV-infected subjects with CD4 counts ≥ 200/mm^3^**. Sputum microbiology was positive in 1 patient with symptoms, 10 with abnormal chest x-ray and 14 with both findings. See Table 2 for details.

Between September 2001 and March 2004 a total of 1794 ambulatory subjects were screened for the DarDar Study. Based on a CD4 count ≥ 200/mm^3 ^and presence of a BCG scar, 1176 subjects proceeded to comprehensive evaluation for active TB (Figure 1). Among these, 136 (12%) subjects were considered ineligible to continue in the Study based on suspect TB and were referred to the NTLP for TB treatment. Figure 1 summarizes the basis for the treatment of presumptive TB among CD4 eligibles. Follow-up data were available on 113 (83%) indicating that all were treated for TB; data on completion of, or response to, treatment were not available since these subjects were ineligible for the main study and did not have detailed follow-up.

Table 2 summarizes the characteristics of patients with and without TB treatment. Patients with TB treatment were more often male, had a lower median CD4 count and a higher rate of both prior TB and a TST ≥ 5 mm. Table 3 summarizes the diagnostic contribution of symptoms, x-ray and microbiology on 136 subjects with TB treatment. An abnormal chest x-ray in the absence of symptoms was the basis for treatment in 68 (50%) cases; 10 of these cases had positive microbiology leaving 58 (43%) cases in which treatment was based on the chest x-ray alone. Symptoms plus an abnormal chest x-ray resulted in treatment for 44 (32%) of cases. Symptoms without a positive chest x-ray resulted in treatment for 11 (8%) of cases.

**Table 2:** Characteristics of HIV-infected patients with CD4 ≥ 200 with and without TB treatment

Category	TB treatment (N = 136)	No TB treatment (N = 1040)	p value
Age, median (interquartile range)	35 (30–40)	33 (27–39)	<0.05
Female N (%)	90 (66%)	775 (75%)	0.049
CD4, median (interquartile range)	319 (248–483)	425 (310–594)	<0.0001
History of TB N (%)*	3/16 (19%)	67/944 (7%)	NS
Tuberculin skin test ≥ 5 mm N (%)*	10/14 (71%)	288/904 (31%)	0.003
Tuberculin skin test ≥ 10 mm*	10/14 (71%)	265/904 (29%)	0.002

* Data only collected on the subjects thought initially to be eligible for the trial; all were later deemed ineligible due to presumptive TB.

**Table 3:** Basis for TB treatment among 1176 HIV-infected subjects with CD4 ≥ 200/mm^3^

Category	No. (%)	Microbiology*			
		
		AFB positive only	Culture positive only	Both positive	Any positive
No TB	1040 (88%)				

TB	136 (12%)	2/124 (2%)	23/106 (22%)	13/106 (12%)	38/136 (28%)
Symptoms (fever or cough ≥ 2 wks)	11/136 (8%)	0/5 (0%)	1/4 (25%)	0/4 (0%)	1/5 (20%)
Abnormal chest x-ray	68/136 (50%)	0/62 (0%)	7/54 (13%)	3/54 (6%)	10/62 (16%)
Symptoms and chest x-ray	44/136 (32%)	2/44 (5%)	6/35 (17%)	6/35 (17%)	14/44 (32%)
Microbiology only	13/136 (10%)	0/13 (0%)	9/13 (69%)	4/13 (31%)	13/13 (100%)

AFB = acid fast bacillus.

* Denominators for microbiology represent proportion in each category who had the given test performed (e.g. 124 subjects had AFB smear performed but only 106 had sputum culture performed).

Among 124 subjects who produced sputum samples 106 were submitted for AFB stain and culture and 18 for AFB stain only. A total of 38 (31%) of 124 tested subjects had a positive AFB smear or mycobacterial culture; these subjects meet a study definition of definite or probable TB (Table 1). Positive sputum microbiology was present in 14 (32%) of 44 subjects tested with both symptoms and an abnormal chest x-ray and in 1 (20%) of 5 subjects tested with symptoms only (p = 0.7). DNA probes were positive for *M. tuberculosis *complex on all 36 isolates tested. Blood cultures were negative on all subjects.

In 13 (10%) of 136 cases of TB the diagnosis was established by sputum microbiology alone in patients with no symptoms and negative chest x-rays. Ten of these cases of "subclinical TB" were reported previously [3]. These patients represent 34% (13 of 38) of all subjects with positive sputum microbiology.

## Discussion

We have shown that 12% of ambulatory HIV-infected patients in Tanzania undergoing comprehensive screening are treated for presumptive and previously undiagnosed active TB. Our results suggest that the optimal diagnosis of TB in this setting requires assessment of both symptomatic and asymptomatic HIV-positive patients using chest x-ray and sputum culture, an observation that differs from consensus international recommendations and has obvious implications for the resources needed to conduct effective and optimal treatment of persons with HIV infection. Confirmation of this preliminary finding will be available when our expert panel completes classification of TB endpoints among the eligible subjects in the trial, including independent review of chest x-rays and response to treatment.

An abnormal chest x-ray in the absence of symptoms was the most common basis for the suspicion of TB. This finding differs from an initial report from Botswana in which an abnormal chest x-ray was very rare among HIV- infected subjects with no symptoms of TB [11] and a study in South Africa where symptom-based screening was effective [12]. However, our results and those of other recent surveys [13,14] including a more recent report from Botswana [15] suggest that the chest x-ray is important in screening and that the standard recommendation to screen for TB based on symptoms alone may be unreliable and insensitive in sub-Saharan Africa [16]. Based on our data, if AFB smear had only been performed on patients with symptoms, 55 patients would have had an AFB smear and only 8 of these (6% of all treated cases) would have been positive and been treated.

Prevalence rates for TB depend on the patient population, screening methods and TB case definitions employed. The 12% point prevalence of TB treatment in our study compares to rates of 3–12% in other studies among HIV- infected persons living in TB endemic countries [12,13,17-22]. A recent study from South Africa using chest x-ray, sputum culture and a clinical definition category for TB found that 11% of subjects in an ART program had previously undiagnosed TB at baseline and that rates of incident TB in those on ART for 3 years continued to be 5–10 fold higher than those for HIV-uninfected persons living in the same region [22].

Microbiologic studies confirmed the diagnosis of TB in 31% of those with suspect disease. Our relatively low rate of positive sputum cultures for TB might have been influenced by overly vigorous decontamination of sputum specimens as suggested by our low sputum culture contamination rate of 1%. However, by routinely performing sputum microbiology in this population, including in those without symptoms, we were able to identify a unique group of subjects with "subclinical TB", i.e., active, culture-positive disease without symptoms (including study definition symptoms as well as absence of additional symptoms such as weight loss or fever <2 weeks) or x-ray findings. A previous report from our study focused on 10 such patients whom we encountered among the initial 500 subjects screened; that report also presented detailed data to eliminate the possibility of false positive cultures and follow-up data to indicate that the early treatment was associated with a more favorable prognosis than typically seen with HIV-associated tuberculosis [3]. The 13 patients in the present series comprise 9% of all patients treated for TB. Rates of subclinical TB might be found to be even higher if more sensitive liquid based media had been used for sputum culture [23]. Investigators in Uganda, India, New York and London have also described HIV-infected subjects with subclinical forms of TB [7,24-26]; in some of these studies "subclinical" includes any active disease without symptoms, regardless of chest x-ray findings.

Sputum culture not only makes an important contribution to the diagnosis of both clinical and subclinical TB in patients with HIV [20,25]. It will also be an increasingly important TB diagnostic tool for another reason: the detection and spread of XDR TB in sub-Sarharan Africa [27]. As we showed previously in Kenya [28] and others have confirmed [5], blood culture for *M. tuberculosis *may make an additional contribution in hospitalized patients with advanced and symptomatic HIV infection but did not contribute to detection of TB in ambulatory subjects with earlier stage HIV infection.

Our overall study is focused on ambulatory HIV-infected persons with CD4 = 200 residing in an area with endemic tuberculosis. We believe our subjects are generally representative of such patients, although there are potential sources of bias. Conceivably, persons with subtle or unreported symptoms of TB might have been preferentially drawn to a TB vaccine trial. Conversely, because all subjects had received BCG at birth, this could have reduced the rate of active TB. In general populations, BCG immunization does not typically affect adult TB rates [29] and among HIV-infected persons limited retrospective data is conflicting [30,31].

Our goal in this report was to describe the frequency and characteristics of subjects who were judged by TB clinicians to require treatment for presumptive TB at the time of screening. Our evaluations were conducted by trained, experienced local physicians, supported by expert radiologic interpretation, albeit by a single radiologist aware of the suspicion of TB. In a study from the United States expert radiologists agreed on chest x-ray suspicion of tuberculosis in 139 subjects (HIV status not stated) approximately 70% of the time, and this suspicion appeared to accurately identify smear negative tuberculosis in 48% of cases [32]. We cannot confirm that all patients treated for TB without microbiologic confirmation had active disease because they were ineligible for our vaccine study and did not have follow-up to assess response to therapy. Current microbiologic methods often fail to confirm disease in subjects who have classic TB symptoms with x-ray changes and subsequent radiologic and clinical response to therapy for TB. In other published series of HIV-associated TB 19–66% of subjects have met a "clinical" definition of TB with negative microbiology [21,33,34].

## Conclusion

Our findings reflect the realities of a presumptive TB diagnosis in regions endemic for both TB and HIV infection. If confirmed in the eligible subjects in our trial whose TB endpoints are classified according to our strict case definitions our findings will have important implications for ART programs in such environments. First, chest x-ray and sputum microbiology will identify a significant number of cases of presumptive TB even among those who deny fever or cough; treatment of these subclinical cases may reduce mortality from HIV-associated TB; thus, an effort should be made to expand availability of these tests in resource poor regions [3]. Second, with rigorous TB screening as many as 12% of ambulatory HIV-infected subjects with CD4 counts = 200/mm^3 ^will qualify for ART based on the decision to treat for TB; early ART in these subjects may reduce mortality [35]. Third, a substantial proportion of ART eligible subjects will require deferral of single drug preventive therapy for latent TB while active TB is being excluded by culture. Finally, these data emphasize the importance of integrating TB and HIV programs in countries with high rates of co-infection, of educating clinicians about the diverse manifestations of TB in HIV-infected patients, and of providing optimal methods for TB diagnosis.

## Competing interests

The author(s) declare that they have no competing interests.

## Authors' contributions

CFvR, RDA, KP, RW, BC and CRH conceived the study. CFvR and MB wrote the first draft of the report and all authors contributed to the final draft. LM, MB and JL were responsible for the clinical conduct of the study. MM directed the microbiologic studies. ST managed the data. CFvR, ST, RDA, LM, and MB participated in the data analysis. BC, and RDA, conducted the statistical analyses. All authors read and approved the final manuscript.

## Pre-publication history

The pre-publication history for this paper can be accessed here:


